# The intermediary mechanism of social fairness perceptions between social capital and farmers’ political participation: Empirical research based on masking and mediating effects

**DOI:** 10.3389/fpsyg.2022.1021313

**Published:** 2023-01-09

**Authors:** Bo Hou, Qiang Liu, Zhiwei Wang, Jing Hou, Suyun Chen

**Affiliations:** ^1^School of Public Administration and Sociology, Jiangsu Normal University, Xuzhou, China; ^2^School of Economics and Management, China University of Mining and Technology, Xuzhou, China; ^3^Research Center of Food Safety and Agricultural Green Development, Jiangsu Normal University, Xuzhou, China

**Keywords:** social capital, social fairness perceptions, farmers’ political participation, mediating effect, masking effect

## Abstract

**Introduction:**

Political scientists have conducted extensive research on the factors influencing political participation, but empirical analyses examining them from the perspective of social fairness perceptions are not common.

**Methods:**

Using large-scale data from the Chinese General Social Survey (CGSS), this study explores the intermediate mechanisms of social fairness perceptions in the influential relationship between social capital and farmers’ diversified political participation based on the structural equation modeling (SEM).

**Results:**

The results show that the positive relationship between social capital and farmers’ political behavior is indirectly influenced by different dimensions of the sense of social fairness. Among them, social trust and social network variables affect political participation mainly through the mediating role of outcome fairness perceptions, while opportunity fairness perceptions significantly widen the gap in political participation between low and high social trust.

**Discussion:**

Therefore, the government should nurture the social capital of rural geo-relational networks and formulate policies based on a social justice perspective inorder to enhance rural residents’ outcome fairness perceptions and increase the political participation of farmers.

## Introduction

As a core element of democratic development in modern societies, political participation not only reflects the behavioral characteristics of individual political socialization but also has essential significance for protecting citizens’ rights and improving governance performance. Modernization has made citizens’ political awareness of participation and rights increasingly advanced. People are eager to have a place in the political arena and to actively influence the political rights system and public political life in some way (Inkeles, 1966). However, recent studies have found that individual political participation rates around the world have generally been decreasing, and the voting rate of Chinese citizens declined by 12.6% points over the decade (Sun and Lei, 2007). In response to this phenomenon, more researchers have begun to explore the factors that influence individual political participation and why individuals’ political participation differs considerably under similar conditions.

The concept of political participation has been enriched by the processes of democratic societies. In the past few decades, the scope of government activity and responsibility has increased in many countries, and the old monolithic view of politics (which saw voting as a key way for the public to influence the political process) is being replaced by a pluralistic view of participation (Van Deth, 2021). Some scholarly studies of Latinos have shown that immigrants are more likely to express their preferences by means other than elections, such as personal contact or seeking the help of government officials (Roman et al., 2021). Whether it is the expression of interests or participation in elections, each individual’s political behavior is inevitably influenced by their social capital. The social network in which an individual is embedded can provide a variety of political and economic information and resources to facilitate political participation, and membership in a party is a prime example of this (Webb et al., 2020). Ideally, the social relations embedded in the various forms of social capital are sufficient to reconcile the interests of different groups. The accumulation of social capital can provide rich social support for the expression of people’s values and interests (Zhong et al., 2022). However, in a transition period in China in which risks are accumulating and becoming concentrated (Hou et al., 2022), differences in the use of capital can deprive farmers of access to information and prevent them from participating equitably in the democratic process. In reality, farmers use a lower level of relational social capital, and the lack of social capital is most likely to lead to a sense of relative deprivation, apathy, and even “voting with their feet” (Ren et al., 2016). Values do not necessarily lead to actions; rather, actions require a factor that links them to values (Lim and Moon, 2022). As a psychological perception factor, social fairness perceptions influence individual preferences and value judgments, and guide political behavior. Therefore, the use of social capital may have an indirect impact on political participation, with social justice as an intermediate variable, but there are few studies on the psychological impact mechanism. Based on this, this study explores the relationship between the use of social capital and Chinese farmers’ political participation, as well as the intermediate mechanisms of social fairness perceptions between them, using data from a national survey.

Different from previous studies, this study expands the model of farmers’ political participation behavior from the perspective of external social capital by introducing variables of perceived opportunity justice and perceived outcome justice, and builds a theoretical model of farmers’ diversified political participation behavior by incorporating psychological factors and external constraint variables into the same analytical framework. Moreover, this study explores whether farmers’ sense of opportunity justice and sense of outcome justice have indirect effects on the relationship between social capital and farmers’ diversified political participation behavior, and further analyzes the indirect effects and identifies which paths can more influence and promote the generation of farmers’ political participation behavior.

## Literature review and research hypotheses

### Social capital and political participation

Social capital refers to the characteristics of social organizations, such as trust, norms, and networks that can improve social efficacy by promoting cooperative behavior. Social capital is often seen as an important factor when studying influences on political participation (Hu, 2008). Leonardi et al. (2001) argues that citizens’ participation is influenced by social capital. When the amount of social capital increases, citizens tend to show more active participation. In research on farmers’ participation in community governance, it was found that dimensions such as social trust and social networks have a significant positive impact on political behavior (Shi et al., 2018). Therefore, it can be considered that social capital has a significant impact on farmers’ political participation. Based on these studies, this paper examines social capital by considering social trust and social networks.

Social trust is a core dimension of social capital, and it refers to the trust that one person or group has in the verbal or written commitment of another person or group. Empirical research shows that social trust has a significant positive impact on urban grassroots election participation (Xing and Luo, 2011), and rural social trust significantly affects village committee election participation. That is, the more trusting the villagers, the more motivated they are to participate in the election (Li, 2016). From this, Hypothesis 1a is proposed:

*H1a*: Social trust has a significant positive impact on farmers’ political participation.

The social network is the basic component of social capital, and the formation of social trust and norms is based on the social network (Pei et al., 2018). As early as 1964, Becker (2009) proposed that social networks can enable citizens to acquire human capital for political participation and therefore promote citizens’ political participation. A differential pattern characterizes the unique social relationship structure in rural areas, and its social network is linked by kinship and geographical relationships, affecting farmers’ social and behavioral patterns. Rural residents use certain network relationships to communicate, exchange, and share information with network members, and to increase their enthusiasm for political participation. In this case, individual farmers are more likely to participate in political consultations. From this, Hypothesis 1b is proposed:

*H1b*: One’s social network has a significant positive impact on farmers’ political participation.

### Social fairness perceptions and political participation

Social fairness refers to the rational distribution of social-political, economic, and other interests within society, and the public’s subjective perceptions of social (un)fairness is the social fairness perception (Cao et al., 2017). It can be divided into macro fairness perceptions and micro fairness perceptions according to the sensitivity of fairness to the allocation of social resources. Previous studies have mostly judged the impact of social fairness perceptions on political participation at the macro level and studied its significant role as a mediating or moderating variable, but there are no in-depth dimensional analyses. For example, Zheng (2019) reveals the moderating role of social fairness perceptions in political knowledge and individual election participation based on the analysis of 8,635 Chinese citizens’ questionnaires. Yao (2020) found that social fairness perceptions have a significant positive impact on citizens’ political participation based on CGSS data, and the mechanism of deeper fairness perceptions is poorly investigated. Social fairness perceptions are divided into opportunity fairness perceptions and outcome fairness perceptions (Meng, 2012), which are the two most basic dimensions for the public to judge the fairness and reasonableness of resource allocation. Opportunity fairness includes the content of distribution fairness, involving opportunities for people to move upward, narrowing the gap, and the possibility of catching up with others, while outcome fairness focuses on the final result of this social resource allocation process. Research conclusions also differ because many scholars are inconsistent in their operationalization of social fairness perceptions in terms of research design, which requires further analysis.

Outcome fairness perceptions are an individual’s substantial feelings of fairness based on the outcome of resource allocation. From this perspective, a higher sense of outcome fairness is conducive to increasing people’s political participation. According to the relative deprivation theory, those groups who feel the unjust consequences of social reality distributions are more likely to have a strong sense of relative deprivation (Guo, 2001). This sense of relative deprivation caused by unfair treatment is likely to cause public dissatisfaction, resistance, and even disappointment with government work, thus discouraging political participation. Individuals with higher perceptions of outcome fairness are more likely to be satisfied psychologically and with the government’s work and then actively participate in politics. From this, Hypothesis 2a is proposed:

*H2a*: Outcome fairness perceptions have a significant positive impact on farmers’ political participation.

Existing studies have mainly focused on outcome fairness perceptions (Ma and Liu, 2010) and ignore the critical role of opportunity fairness perceptions, not to mention the relationship between them and the impact of different information mechanisms on political participation. Research has also shown that people tend to pay more attention to opportunity fairness than to outcome fairness (Trautmann and van de Kuilen, 2016; Hou, 2020). In particular, the increased marketization of Chinese society has led to an increase in outcome fairness perceptions and a decrease in opportunity fairness perceptions among the public (Xu et al., 2020). Some scholars have found that individual perceptions of fairness and justice weaken the relationship between political socialization and political participation. That is, groups with a lower level of perception of fairness and justice experience a more substantial and more apparent positive effect of political socialization (such as civic skills) on political participation. That is, individuals with lower opportunity fairness perceptions are more motivated to enhance their political behavior (Neufeind et al., 2014). From the perspective of social mobility, the “winners” and “losers” in social transformation have very different attitudes toward distribution fairness. For example, Whyte (2012) reveals farmers and residents of western China are more inclined to accept the current inequalities, but they find it difficult to participate meaningfully in political life due to their limited social resources. People with resource advantages will feel the situation deteriorate if they find the same development opportunities as the disadvantaged groups, so they will generate a lower opportunity fairness perception and increase the possibilities for political participation by increasing access to relevant government departments. From this, Hypothesis 2b is proposed:

*H2b*: Opportunity fairness perceptions have a significant negative impact on farmers’ political participation.

### Social capital and social fairness perceptions

Although few studies have directly pointed out the specific relationship between social capital and farmers’ social fairness perceptions, relevant work also reflects that higher social capital leads to a high sense of social fairness. From different dimensions of social capital, the higher the degree of trust in strangers and organizations, the higher the degree of social participation, and the higher the level of social fairness (Xu and Liu, 2018). Next, this paper will identify the internal connection with social fairness perceptions from the two dimensions of social trust and social networks.

Empirical studies have found a positive correlation between social fairness perceptions and trust (Hu, 2013). However, most of the above research focuses on the impact of social fairness perceptions on trust and finds that individuals who have higher social fairness perceptions tend to trust the majority of society. Regarding the impact of social trust on fairness perceptions, some scholars have found that the more trust an individual has in strangers, different professions, and (informal) organizations, the higher their social fairness perceptions (Xu and Liu, 2018). Given the positive correlation between these variables and the positive impact of social trust on social fairness perceptions, it can be considered that social trust has a significant positive impact on farmers’ social fairness perceptions. Accordingly, Hypotheses 3a and 3b are proposed:

*H3a*: Social trust has a significant positive impact on farmers’ opportunity fairness perceptions.

*H3b*: Social trust has a significant positive impact on farmers’ outcome fairness perceptions.

Theoretical studies have found that social networks and social fairness perceptions are closely related. Hansen (2009) uses social network theory to analyze the unfair social power network and the imbalance of social capital, and believes that social unfairness can be solved by constructing and managing social networks. Although unfairness and unfairness perceptions are not the same concept, low income brought about by social unfairness can significantly affect an individual’s social fairness perceptions. Sociologist Durkheim (2019) also argued that individuals cooperate with each other in social networks to form a sense of community, and people will pay more attention to collective interests rather than to individual interests, so the sense of unfairness will be weakened. Accordingly, Hypotheses 4a and 4b are proposed:

*H4a*: Social networks have a significant positive impact on farmers’ opportunity fairness perceptions.

*H4b*: Social networks have a significant positive impact on farmers’ outcome fairness perception.

### The mediating effect of social fairness

Among the research on the factors that influence political participation, there are few findings related to the sense of social fairness perceptions as a mediating variable, while there is richer research on its role as a moderating variable on the influence of other factors on political participation. For example, social fairness perceptions play a moderating role in the influence of political trust on political participation. Specifically, the higher the level of social fairness perceptions, the stronger the influence of political trust on political participation (Zheng, 2013). Social fairness perception is also a boundary variable of political knowledge and political participation, and it positively moderates the influence of political knowledge on citizens’ electoral participation (Zheng, 2019).

However, the above research has motivated this paper to consider the “social capital—social fairness perception—political participation” pathways. The literature review shows that social capital significantly affects social fairness perceptions and farmers’ political participation, and social fairness perceptions can also have a significant positive impact on farmers’ political participation. Therefore, this paper asserts that social capital can not only directly affect farmers’ political participation but also indirectly affect it through social fairness perceptions. That is, social fairness perceptions play an intermediary role in social capital and farmers’ political participation. Accordingly, Hypotheses 5 and 6 are proposed:

*H5*: Social fairness perceptions have a positive mediating effect between social trust and farmers’ political participation.

*H6*: Social fairness perceptions have a positive mediating effect between social networks and farmers’ political participation.

**Figure 1:**
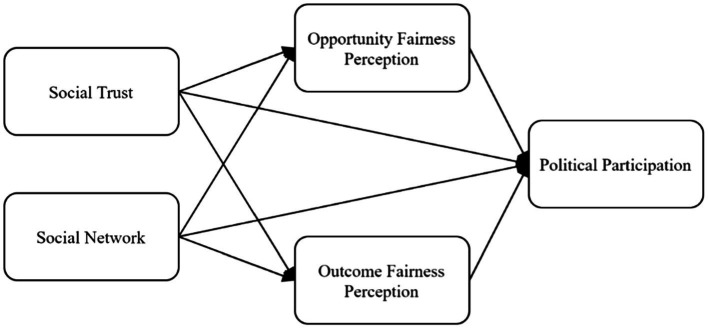
. The influence mechanism of farmers’ political participation.

Based on the above theories and hypotheses, this paper constructs the theoretical framework of the influence mechanism of farmers’ political participation (Figure 1).

## Materials and methods

### Data sources and sample composition

The CGSS is the earliest nationwide, comprehensive, and continuous large-scale academic survey project in China. The survey is jointly implemented by the Renmin University of China and academic institutions across the country. It is administered to a sample of households and inquiries about topics including personal information, family structure, lifestyle, and social participation. This paper uses data released by the institution in 2018. For that year, the data set included a total of 10,968 valid questionnaires covering 478 villages in 28 provinces/municipalities/autonomous regions in China. As the authoritative data source for studying social issues in China, the CGSS provides authentic and comprehensive data for research on China in many fields, and represents high academic research value. Compared with the questionnaires and data released in other years, the social fairness perception module included in the CGSS questionnaire released in 2018 is more representative and comprehensive for the research on the intermediate mechanism between social capital and farmers’ political participation, making it suitable for this study. According to the needs of the study, the sections on socio-economic status, living conditions of adult farmers, and political participation were selected, and 1,514 valid cases were obtained after the core variables were processed for missing data and outliers. The demographic characteristics of the sample are shown in Table 1.

**Table 1:** Demographic characteristics of the sample.

**Variable**	**Attribute**	**Number**	**Frequency**	**Variable**	**Attribute**	**Number**	**Frequency**
*Gender*	Male	728	48.1	*Political status*	Party member	77	5
	Female	786	51.9		Non-party member	1,437	95
*Nationality*	Han	1,370	90.5	*Marital status*	Single/cohabitation	152	10
	Minority	144	9.5		Married or otherwise	1,362	90
*Age*	18–44 years	590	39	*Education*	Elementary school and below	728	48.1
					Junior high school	518	34.2
	45–59 years	491	32.4		High school	175	11.5
	≥60 years	433	28.6		Junior college	60	4
					Undergraduate and above	33	2.2

### Variable selection

This study uses the relevant questions in the CGSS questionnaire released in 2018, combined with the measurement methods of latent variables as described in the literature review. It divides farmers’ political participation into two dimensions: voting and appeals (Xu and Hu, 2011). Social fairness perceptions are divided into opportunity fairness perceptions and outcome fairness perceptions (Xu et al., 2020). Social capital is divided into the social network and social trust based on the interpersonal network perspective (Leonardi et al., 2001). The specific operational indicators, assignments, and descriptions are shown in Table 2.

**Table 2:** Meaning, assignment, and description of variable operational indicators.

**Variable**	**Variable definitions**	**Assignment description**	**Mean**	**SD**
**Political participation**				
Voting type	Did you vote in the last village committee election?	No = 0; yes = 1	0.55	0.498
Appealing type	How effective do you think the administrative authorities have been in resolving/going to court/writing petitions?	Fully invalid = 1; less valid = 2; general = 3; usually valid = 4; always valid = 5	3.26	0.675
**Social fairness perception**				
Opportunity fairness perception	As long as children are hardworking and smart enough, they will have the same chance of further education	Strongly disagree = 1; disagree = 2; indifferent = 3; agree = 4; Strongly agree = 5	3.83	0.896
	In our society, the descendants of workers and farmers have just as much chance of becoming wealthy and established as the descendants of others	Strongly disagree = 1; disagree = 2; indifferent = 3; agree = 4; Strongly agree = 5	3.58	1.037
Outcome fairness perception	More taxes should be levied from the rich to help the poor	Strongly disagree = 1; disagree = 2; indifferent = 3; agree = 4; Strongly agree = 5	3.83	0.894
	Now some people earn more and some less, but that is fair	Strongly disagree = 1; disagree = 2; indifferent = 3; agree = 4; Strongly agree = 5	3.61	0.976
	In general, do you think society today is fair or unfair?	Totally unfair = 1; compare unfair = 2; indifferent = 3; compare fair = 4; totally fair = 5	3.22	1.003
**Social capital**				
Social trust	In general, do you agree that most people in this society are trustworthy?	Strongly disagree = 1; compare disagree = 2; had to say = 3; compare agree = 4; Strongly agree = 5	3.51	0.941
	In general, do you agree that in this society, if you are not careful, others will try to take advantage of you?	Strongly agree = 1; compare agree = 2; had to say = 3; compare disagree = 4; Strongly disagree = 5	2.99	1.012
	How familiar are you with your neighbors/other residents of the same village?	Very unfamiliar = 1; less familiar = 2; general = 3; more familiar = 4; very familiar = 5	4.05	0.968
Social network	In the past year, have you often socialized/visited in your free time?	Never = 1; rarely = 2; sometimes = 3; often = 4; very often = 5	2.94	0.916
	How often do you socialize with your neighbors?	Never = 1; once a year or less = 2; several times a year = 3; once a month = 4; several times a month = 5; once or twice a week = 6; almost every day = 7	2.99	1.826
	How often do you socialize with other friends?	Never = 1; once a year or less = 2; several times a year = 3; once a month = 4; several times a month = 5; once or twice a week = 6; almost every day = 7	3.65	1.862

### Model construction

This paper exploresthe the intermediary mechanism of social fairness perceptions between social capital and farmers’ political participation.This analysis has the basic characteristics of unavoidable subjective errors, therefore, we usestructural equation modeling (SEM) for the empirical analysis. The SEM measurement model reflects the relationship between latent and observed variables, and thestructural model reflects the relationship between latent variables. SEM can be represented by three matrix equations:


(1) X=Λxξ+δ

(2) Y=Λyη+ε

(3) η=Bη+Γξ+ζ

Equations (1) and (2) are the measurement models, and Equation (3) is the structural model, where η and ξ are endogenous latent variable and exogenouslatent variable matrices, respectively; Λx and Λy are the relationship coefficient matrices of observed variables X and Y, respectively; δ, ε, and ζ represent residual matrices; η is determined by B and the Γ coefficient matrix; and the error term ζ establishes a relationship between the endogenous latent variables and the exogenous latent variables to construct the SEM. This study specifically includes 5 latent variables and 12 observed variables.

## Results

Before data analysis, the measurement model should be tested to ensure the scientificity and accuracy of the structural equation modeling. So it can be determined to explore the space for further optimization according to the relevant correction indicators, and the relevant indicators can be output for basic testing. Mediated validity tests are conducted to verify the research hypotheses presented in the previous section based on the standardized path coefficients of the modified structural equation modeling.

### Model Fit study

The model fit index indicates the degree of consistency between the sample covariance matrix and the model set by the researcher, including fixed parameters and free parameters, with a better fit index indicating that the research model is more consistent with the sample data. In this study, GFI, IFI, PGFI, RMSEA, and other fitness indicators were selected to comprehensively test the model’s fit (Table 3). Except for the observation that CMIN and IFI are close to the fitting results caused by large samples, all other indicators reached the ideal standard, indicating that the model’s overall fit meets the requirements, and the results of the parameter estimation are credible. The SEM design diagram in Figure 1 is supported.

**Table 3:** Model fit report table.

**Indicators**	**Meaning**	**Value**	**Std.**	**Results**
GFI	Goodness of fit index	0.956	> 0. 90	Ideal
IFI	Incremental fit index	0.827	> 0. 90	Near
PGFI	Parsimonous goodness-fit index	0.589	> 0. 50	Ideal
RMSEA	Root mean square error of approximation	0.068	< 0. 1	Ideal
PCFI	Parsimonious comparative-fit-index	0.592	> 0. 50	Ideal
PNFI	Parsimonious normed fit index	0.579	> 0. 50	Ideal
CN	Critical sample size	1,514	> 200	Ideal
CMIN	Sample chi-square value	443.9	The smaller the better	Near

### Structural path test

The path analysis stage begins after passing the test of the overall fit of the model. This study used the maximum likelihood method to infer and test the study hypotheses, with Amos 27.0 being used for the SEM analysis. Table 4 lists the path coefficients, standard errors, critical ratio values, and significance of the farmers’ political participation model.

**Table 4:** Structural path test results.

**Path coefficient**			**Unstd.**	**S.E.**	***T*-value**	***P***	**Std.**
Opportunity fairness perception	←	Social trust	1.667	0.209	7.988	***	0.99
Opportunity fairness perception	←	Social trust	1.667	0.209	7.988	***	0.99
Outcome fairness perception	←	Social trust	1.967	0.303	6.486	***	2.545
Opportunity fairness perception	←	Social network	0.268	0.047	5.738	***	0.65
Outcome fairness perception	←	Social network	0.336	0.064	5.262	***	1.774
Political participation	←	Social trust	0.557	0.191	2.917	0.004	1.131
Political participation	←	Social network	0.068	0.034	1.991	0.046	0.561
Political participation	←	Opportunity fairness perception	−0.32	0.099	−3.226	0.001	−1.093
Political participation	←	Outcome fairness perception	0.464	0.132	3.516	***	0.729

*, **, and *** denote significance levels of 0.10, 0.05, and 0.01, respectively.

### Social capital and farmers’ political participation

The regression coefficient of social trust on farmers’ political participation was 1.131 and significant at the confidence level of α = 0.05. This suggests that H1a is supported. That is, the higher the degree of trust of the farmers in the people around them, the higher probability of their participation in politics, consistent with previous studies (Xing and Luo, 2011; Li, 2016). When the trust level among villagers is high, information sharing and communication opportunities increase and the cost of farmers’ political participation is correspondingly reduced, thus increasing the political participation rate. In addition, considering the existence of the principal–agent relationship between voters and candidates, farmers not only pay attention to their interests when engaging in political participation (here, this refers to their election participation), but also consider the impact of current political participation on the entire village to prevent the moral hazard problem caused by information asymmetry under the principal–agent relationship. When the level of social trust among villagers increases, they are more likely to consider the welfare and benefits of others when engaging in political acts. When self-interest exists and altruism increases, political participation as an act of expressing demands and influencing decisions becomes more necessary for farmers, and the political participation rate increases accordingly.

The regression coefficient of social networks on farmers’ orderly political participation was 0.561 and significant at the confidence level of α = 0.05. Therefore, H1b is supported. That is, the richer the villagers’ social network resources, the more likely they will be to participate in voting and appealing to political leaders. Work by Pei et al. (2018), Lu and Zhu (2016), and others has reached similar conclusions. Pei Zhijun found that social networks play a positive moderating role in the influence of political efficacy on participation in public consultations and can strengthen the positive relationship between them. Lu Chuntian found that vertical social networks play a positive mediating role in rural youths’ participation in public affairs. This is because social networks provide farmers with more access to political information, thus increasing their ability and willingness to participate in politics. Individuals involved in the complex social networks tend to expect to make rational choices that influence government behavior, while those lacking social interaction and connections are often excluded from political information, limiting their ability to participate in politics to some extent (McLeod et al., 1999). The research shows that individuals at the center of social networks and occupying dense resources are more eager to express their will and are thus more active in collective action (Su and Feng, 2013).

### Social fairness perceptions and farmers’ political participation

The regression coefficient of opportunity fairness perceptions on farmers’ orderly political participation was −1.093 and significant at the confidence level of α = 0.01, H2b is supported. That is, the stronger the villagers’ opportunity fairness perceptions, the less likely they are to participate in voting and appealing behaviors. Studies have found that residents in more developed areas are less willing to respond to policies than those in less developed areas (Song et al., 2022). This is related to the unequal distribution of public resources, unfair labor employment opportunities, and weak political expression discourse in China today. As a principle of distribution, villagers who perceive that they are being treated unequally in this resource distribution process are more likely to appeal to the relevant institutions to participate in political actions and to draw on the support of the state and social forces to safeguard their rights and interests. In other words, in today’s information age, individuals who perceive opportunities to be unfair are more likely to be activists and to engage in political participation. They not only pay attention to income but also (and more so) to the fairness of the distribution of opportunities.

The regression coefficient of outcome fairness perceptions on farmers’ orderly political participation was 0.729 and significant at the confidence level of α = 0.01, H2a is supported. Although previous authors have not directly studied the association between these variables, relevant empirical studies can also reasonably explain the relationship. For example, research shows that low income caused by unfairness will provide individuals with fewer resources for political participation, which will lead them to think that political participation is unreachable for them or that even if they participate, the results are futile (Uslaner and Brown, 2005). Furthermore, the unequal distribution of economic resources can reduce the motivation of low-income people to participate in political life, and this group tends to show lower levels of social fairness perceptions (Ma and Li, 2012). As a result, individuals with higher social fairness perceptions have a higher rate of political participation.

### Mediating/masking effect test

SEM includes correlation analysis, factor analysis, and regression analysis. It not only deals with the path relationship between latent variables and explicit variables but also observes the magnitude and direction of direct effects, indirect effects, and the total effect (as shown in Table 5), which is a question that cannot be answered by traditional regression analysis. According to the mediating effect test procedure proposed by the econometrician Wen and Ye (2014), the path is first divided into two groups. Path analysis is then performed on the independent variable X and the dependent variable Y, the independent variable X and the mediating variable M, and the mediating variable M and the dependent variable Y. Finally, the significance levels of the path coefficients are tested.

**Table 5:** Results of the mediating effect test.

Regression equation	Social Trust (X_1_)		*Y* = cX_1_ + ex_1_		Social Network (X_2_)		*Y* = cX_2_ + ex_1_	
	Social fairness perception (M)		*M* = aX_1_ + ex_2_		Social fairness perception (M)		*M* = aX_2_ + ex_2_	
	Political participation (*Y*)		*Y* = c’X_1_ + bM + ex_3_		Political participation (*Y*)		*Y* = c’X_2_ + bM + ex_3_	
Coef.	c	a	b	c’	c	a	b	c’
Sig.	0.915	0.579, 1.681	−0.360, 0.349	0.537	0.212	0.077, 0.136	0.090, 0.646	0.117
	0.003	0.000, 0.000	0.016, 0.006	0	0	0.000, 0.016	0.295, 0.000	0.095
*T*-value	2.965	6.041, 4.866	−2.409, 2.762	3.584	3.401	3.401, 2.402	1.048, 4.617	1.67
Type	Partial mediation (ab/c = 41.3%)				Complete mediation			

**Figure 2:**
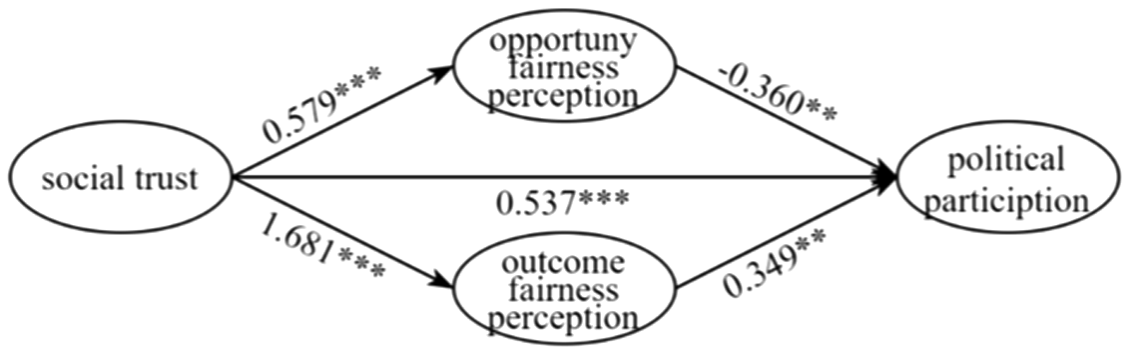
Coefficient of the transmission path of the mediating effect of social trust.

As shown in Figure 2, this transmission path can be classified as a mediating effect because the total effect c of the independent variable X on the dependent variable Y is significant in the impact of social trust on political participation. The indirect effect is significant because the regression coefficients a and b in the two models successfully reach significance. The direct effect is significant when the regression coefficient c’ 0.05 is tested. The results include the absolute value of the ratio of the indirect effect to the direct effect (|ab/c’|), which is equal to 0.228, because the path of action of the sense of opportunity fairness in ab and c’ is of a different sign. According to MacKinnon et al.’s (2000) distinction between mediating effects and masking effects, the nature of the indirect effect of opportunity fairness perceptions on social trust and political participation is a “masking effect” rather than a “mediating effect.” This means that controlling opportunity fairness perceptions will significantly expand the difference in political participation behavior between high and low social trust. This not only verifies H3a, but also indicates that the unique geopolitical structure of rural society establishes a stable trust mechanism, and mutual trust among villagers facilitates the exchange and sharing of information among individuals, increasing their demand for openness in the participation process. When there is a lack of trust between villagers, unfairness perceptions motivate people to participate in election activities to ensure that they express their interests through political behavior. The mediating effect ratio of ab/c is 0.641 while ab and c’ are equal in the role path of outcome fairness perceptions. This suggests that the indirect effects of outcome fairness perceptions on social trust and political participation are partly mediating effects, and increasing farmers’ social trust and outcome fairness perceptions will significantly impact their political participation. H3b is supported. Mutual trust among villagers can strengthen social identity and a sense of shared responsibility for participation, enhance villagers’ outcome fairness perceptions, and further increase the ability of village members to participate in political activities such as elections.

Therefore, under the dual effects of opportunity fairness and outcome fairness, social fairness perceptions play a partial mediating role, with an indirect effect of 41.3%. H5 is supported. Previous studies have found that democratic values can promote the transformation of educational resources into political trust (Kołczyńska, 2020), while social fairness perceptions can promote the influence of political trust on people’s electoral participation (Zheng and Zhao, 2020). These studies have been expanded here, and we find that social fairness perceptions can also promote the influence of social trust on people’s diversified political participation. This is due to the close relationship between social trust and political trust (Schyns and Koop, 2010). Higher social trust is the cornerstone of political trust, so individual universal trust can lead to positive political participation.

**Figure 3:**
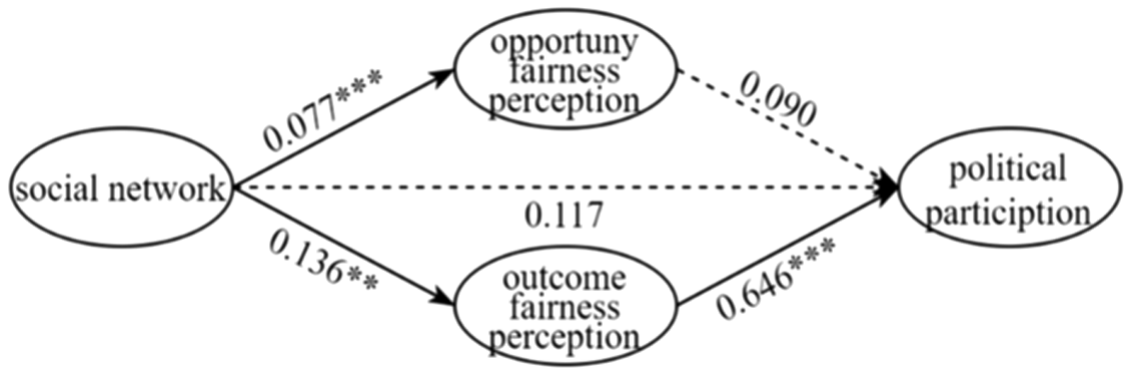
Coefficient of the transmission path of the mediating effect of social network.

The regression coefficients of social network on opportunity fairness perception and outcome fairness perception were 0.077 and 0.136, respectively, and significant at the confidence level of α = 0.01, H4a and H4b are supported. The direct effect c’ is not significant, indicating the existence of a mediating effect in the two paths of social networks as they act on political participation (Figure 3). Further research found that opportunity fairness perceptions did not pass the significance test in the mediating path of social networks–political participation, while outcome fairness perceptions played a fully mediating role between social networks and political participation. The inclusion of the social fairness perceptions variable changes the pathway of villagers’ participation networks, affecting political participation activities by influencing outcome fairness perceptions among members. H6 is supported.

Similar conclusions have been found in research on Chinese rural endowment insurance participation. The results show that outcome fairness perceptions have a significant positive promotion effect, while opportunity fairness perceptions have no statistically significant effect (Zheng and Huang, 2020). This study confirms the conclusion that social fairness perceptions have a positive effect on individual government cooperation behavior from the perspective of social networks. The denser the villagers’ participation network, the more likely they will be to cooperate for the common good. The unique participation network of rural society increases the potential cost of deception in people’s individual transactions, fosters strong reciprocity norms, forms a high sense of outcome fairness, increases the probability of network members being mobilized, and thus leads to higher levels of political participation among network members. In summary, the empirical findings reveal the path of the “social capital–social fairness perceptions–political participation” relationship, in which social fairness perceptions play a partially mediating role in the influence of social trust on political participation, and a complete mediating role in the influence of social networks on political participation.

## Conclusion and implications

This paper focuses on the issue of farmers’ political participation and examines in depth the influence mechanisms of social capital and social fairness perceptions on farmers’ political participation based on data from the CGSS. The findings show that social trust, social networks, and outcome fairness perceptions have a significant positive effect on farmers’ political participation, while opportunity fairness perceptions have a significant negative effect on political participation. The results further show that there are two mechanisms of a “masking effect” and “mediating effect” between the impact of social fairness perceptions and social capital on farmers’ political participation. Specifically, in the impact of social trust on political participation, the mechanism of opportunity fairness perceptions is the masking effect, and the outcome fairness perceptions have a strong partial mediating effect. In the impact of social networks on political participation, opportunity fairness perceptions have no significant effect, and outcome fairness perceptions show a complete mediating effect.

Based on the above research, this paper has the following implications. First, it is necessary to transform the existing social capital stock in rural areas, explore and utilize traditional social capital, build a multi-dimensional social network, and enhance mutual trust among rural residents. We should also make full use of the collective role of elites in the village, and utilize their charisma and influence to build consensus on villagers’ participation, thereby promoting the development and improvement of the village co-governance model. The second implication of this research is that it is necessary to cultivate the civic spirit, nurture rural organizations, develop community-style social capital, increase mutual trust among members, form strong interactions with the government, and participate in the management of public affairs together. We should cultivate social capital for villagers’ political participation by holding characteristic cultural activities to pay attention to the reasonable demands of villagers, creating mutually beneficial social customs to enhance their senses of responsibility and collectivity. Third, the government should deepen the reform of the income distribution system, optimize the income distribution results, strive to ensure that the fruits of development are shared by more people, and firmly follow the path of common prosperity. It should also ensure the participants’ right to know about and participate in the system and procedures, improve the transparency of the election process, and alleviate villagers’ feelings of unfairness, as well as focus on fully embodying the idea of people-oriented development in education and life; promote fairness in income distribution, medical services, and employment opportunities; formulate policies based on a vision of social justice; improve the fairness of the distribution of social benefits; and reduce the differentiation of various social interest groups. At the same time, in terms of maintaining and rebuilding social trust, the government needs to take different countermeasures for groups with different levels of trust so that villagers can truly perceive fairness and justice in village governance, so as to enhance the political participation of different farmers’ groups.

Although the theoretical hypothesis and model setting have been explained and demonstrated in detail in this study, there are still areas that need to be supplemented and improved. Constrained by the year difference in the setting of the questionnaires, this study did not include the time dimension to specifically examine the impact of social fairness perceptions on farmers’ diversified political participation in different periods of social development. Therefore, it is impossible to analyze the impact of changes in the social environment on people’s political participation. In addition, the age, education, culture, and other demographic characteristics of individuals will affect the social role and status of farmers to a certain extent, thereby affecting the political participation of farmers. However, this study did not control for some relevant personal and cultural variables. Future scholars who wish to explore the influence mechanism of other non-economic factors on farmers’ political participation can introduce individual and cultural variables to expand the theoretical model for subsequent research.

## Data availability statement

The original contributions presented in the study are included in the article/supplementary material, further inquiries can be directed to the corresponding author.

## Author contributions

BH and QL: have performed the original research work, evolution of overarching research goals and aims, and development or design of methodology. ZW: committed to study concept, model, software, and language correction of the manuscript. JH: have contributed in revising manuscript, polishing the English language, as well as providing funding for research. SC: have contributed in literature review. All authors contributed to the article and approved the submitted version.

## Conflict of interest

The authors declare that the research was conducted in the absence of any commercial or financial relationships that could be construed as a potential conflict of interest.

## Publisher’s note

All claims expressed in this article are solely those of the authors and do not necessarily represent those of their affiliated organizations, or those of the publisher, the editors and the reviewers. Any product that may be evaluated in this article, or claim that may be made by its manufacturer, is not guaranteed or endorsed by the publisher.
